# Combined therapy of bilateral transcranial direct current stimulation and ocular occlusion improves visual function in adults with amblyopia, a randomized pilot study

**DOI:** 10.3389/fnhum.2023.1056432

**Published:** 2023-02-03

**Authors:** Raul Castillo-Astorga, Lucia Del Valle-Batalla, Juan José Mariman, Ivan Plaza-Rosales, Maria de los Angeles Juricic, Pedro Esteban Maldonado, Marlene Vogel, Romulo Fuentes-Flores

**Affiliations:** ^1^Escuela de Medicina, Facultad de Medicina, Universidad de Chile, Santiago, Chile; ^2^Departamento de Kinesiología, Facultad de Medicina, Universidad de Chile, Santiago, Chile; ^3^Departamento de Kinesiología, Facultad de Artes y Educación Física, Universidad Metropolitana de Ciencias de la Educación, Santiago, Chile; ^4^Núcleo de Bienestar y Desarrollo Humano, Centro de Investigación en Educación (CIE-UMCE), Universidad Metropolitana de Ciencias de la Educación, Santiago, Chile; ^5^Departamento de Neurociencia, Facultad de Medicina, Universidad de Chile, Santiago, Chile; ^6^Departamento de Tecnología Médica, Facultad de Medicina, Universidad de Chile, Santiago, Chile; ^7^Biomedical Neuroscience Institute (BNI), Facultad de Medicina, Universidad de Chile, Santiago, Chile; ^8^Departamento de Oftalmología, Facultad de Medicina, Universidad de Chile, Santiago, Chile; ^9^Servicio de Oftalmología, Hospital Exequiel González, Santiago, Chile; ^10^Servicio de Oftalmología, Hospital Clínico de la Universidad de Chile, Santiago, Chile

**Keywords:** tDCS, amblyopia, adults, clinical trial, occlusion therapy, neuroplasticity, visual acuity, visual evoked potentials

## Abstract

**Background:**

Amblyopia is the interocular visual acuity difference of two lines or more with the best correction in both eyes. It is treated with ocular occlusion therapy, but its success depends on neuroplasticity, and thus is effective in children but not adults. Transcranial Direct Current Stimulation (tDCS) is suggested to increase neuroplasticity.

**Objective:**

To determine if combined intervention of bilateral tDCS and ocular occlusion improves visual function in adults with amblyopia.

**Methods:**

A double-blind randomized, controlled pilot trial was conducted in 10 volunteers with amblyopia. While applying ocular occlusion and performing a reading task, participants received bilateral tDCS (*n* = 5) or sham stimulation (*n* = 5), with the anodal tDCS electrode in the contralateral visual cortex and the cathodal in the ipsilateral visual cortex in relation to the amblyopic eye. Visual function (through visual acuity, stereopsis, and contrast sensitivity tests) and visual evoked potential (with checkerboard pattern stimuli presentation) were evaluated immediately after.

**Results:**

A total of 30 min after treatment with bilateral tDCS, visual acuity improved by 0.16 (± 0.025) LogMAR in the treatment group compared with no improvement (–0.02 ± 0.02) in five controls (*p* = 0.0079), along with a significant increase in the amplitude of visual evoked potentials of the amblyopic eye response (*p* = 0.0286). No significant changes were observed in stereopsis and contrast sensitivity. No volunteer reported any harm derived from the intervention.

**Conclusion:**

Our study is the first to combine anodal and cathodal tDCS for the treatment of amblyopia, showing transient improved visual acuity in amblyopic adults.

## 1. Introduction

Amblyopia is a vision disorder defined as the reduction, either in one or both eyes, of the best-corrected visual acuity (BCVA), in the presence of a structural or functional eye abnormality that cannot completely justify such a visual acuity loss. Clinically, amblyopia is defined as the presence of a BCVA on either eye of 20/40 (>0.3 LogMAR) or a BCVA difference between both eyes of two or more lines, either in Snellen or LogMAR charts (Blair et al., 2020). It is a major cause of visual dysfunction affecting about 99.2 million people worldwide, and it is expected to grow up to 172.2 million in 2030. A meta-analysis described a global prevalence of 1.44%, being higher in Europe (2.9%) and North America (2.41%) than in Asia (1.09%), and Africa (0.72%) (Fu et al., 2020).

Amblyopia develops at the beginning of life because of any condition that could lead to a decrease in the activation of the visual pathway of the amblyopic eye, resulting in a fellow eye’s increased representation in the primary visual cortex. The current gold standard treatment for amblyopia is to deliver refractive correction (Writing Committee for the Pediatric Eye Disease Investigator Group, Cotter et al., 2012) and then use occlusion or penalization therapies (using blurring eye drops) in the fellow eye, thus forcing the use of the amblyopic eye (Clarke, 2007; Loudon and Simonsz, 2007). However, the effectiveness of this treatment depends largely on the patient’s neuroplasticity, that is, on the brain’s ability to change functionally and structurally in response to experience (von Bernhardi et al., 2017). Therefore, a drastic reduction in the effectiveness of occlusion therapy occurs in the age group over 9 years, in which neuroplasticity of the visual pathway is significantly decreased (Barrett et al., 2004). Unfortunately, the higher prevalence of amblyopia is found in people above 20 years (3.29%), in which occlusion treatment, the most utilized strategy, has shown to be less effective (Fu et al., 2020). Given that the efficacy of ocular occlusion therapy decreases progressively with age and significantly above 17 years of age (Barrett et al., 2004), it is necessary to find new alternatives for those diagnosed later. One possible solution is to combine ocular occlusion therapy with a method that increases neuroplasticity, such as transcranial Direct Current Stimulation (tDCS).

Transcranial direct current stimulation is one of the most used forms of non-invasive brain stimulation and consists of injecting low-intensity continuous current to the brain with surface electrodes. This procedure does not cause the neurons to fire action potentials but modulates the excitability through sub-threshold bidirectional changes of the resting membrane potentials (Nitsche et al., 2004). The most used protocols for tDCS recommend at least 20 min of stimulation to ensure increased neuroplasticity, which lasts from 1.5 to 4 h immediately after the stimulation (Nitsche et al., 2004; Thair et al., 2017). The clinical application of tDCS has been proven to be safe and well-tolerated, and no severe adverse effects have been reported so far (Antal et al., 2017). A minimum of two electrodes are needed for the current to flow, and the direction of this current determines whether an electrode is an anode or a cathode.

Two of the tDCS protocols are relevant to this study: unilateral and bilateral tDCS. Unilateral tDCS refers to placing the anode over the target area while the cathode is placed elsewhere in a non-stimulable area (contralateral supraorbital region, cheek, neck, or arm generally). In bilateral tDCS, the anode and cathode are located contralaterally in both right and left target areas of the skull (Halakoo et al., 2020), Anodal tDCS is often referred to as “activating” tDCS, because it increases the membrane resting potential, increasing the spontaneous firing activity. Cathodal tDCS is referred to as “suppressing” tDCS, since it lowers the membrane resting potential, decreasing the spontaneous firing activity (Stagg and Nitsche, 2011).

Few studies use tDCS as a therapeutic approach for amblyopia, and they generally showed clinical improvements in visual function. The first study combined the treatment of dichoptic video games with anodal tDCS to the visual cortex (with the active electrode placed in the occipital midline) and stimulating during the first 15 min of each session, showing an improvement in stereopsis and visual acuity that stayed above basal measurements for at least 3 months after the intervention when compared to dichoptic video games alone (Spiegel et al., 2013b). Also, anodal tDCS of the visual cortex transiently increased contrast sensitivity and visual evoked potentials (VEPs) amplitudes in the amblyopic eyes compared to the control eyes (Spiegel et al., 2013b). As the activating effect of anodal tDCS lies in its ability to reduce the local concentration of GABA (Stagg et al., 2009; Halakoo et al., 2020) thus changing functional connectivity (Bachtiar et al., 2015), these results can be explained by a reduction in the inhibitory effect from the fellow eye over the amblyopic eye. A study that compared anodal and cathodal tDCS applied to the primary visual cortex (V1), found that while anodal stimulation transiently increased VEP amplitudes and contrast sensitivity for amblyopic eyes, cathodal stimulation decreased both (Ding et al., 2016). The fact that cathodal tDCS has an inhibitory effect based on its ability to reduce local glutamate concentration (Stagg et al., 2009; Clark et al., 2011) might explain these results, yet, when cathodal tDCS is applied unilaterally to the V1 contralateral to the amblyopic eye, it improves visual acuity and increases the amplitude of VEPs on the ipsilateral visual cortex (Bocci et al., 2018), suggesting that unilateral stimulation to a single hemisphere might be key for the therapeutic effect. The improved visual acuity and the increased VEP in the ipsilateral non-stimulated cortex observed in this study can be explained by the suppression in the stimulated cortex of the transcallosal excitatory neurons, which project to the GABAergic neurons of the non-stimulated cortex (Palmer et al., 2012). Thus, the suppressing effect of cathodal tDCS applied to one cortex will decrease inhibition in the contralateral cortex.

Given the correct neurodevelopment of the ocular and visual pathways during the early critical periods, the inputs from both eyes are represented in almost the same proportion in each primary visual cortex in the mature brain (14–15). In a cat model, preventing natural stimulation of a single eye by monocular occlusion during early postnatal life results in a dramatic reduction of the visual cortex neurons responding to stimulation of the occluded eye (14). Comparably, fMRI studies show that humans with amblyopia have a clear dominance of the fellow over the amblyopic eye in V1 (Hubel and Wiesel, 1970; Liu et al., 2002, 2004). In turn, the levels of eye dominance are correlated to the difference in GABAergic inhibition between the eyes during visual processing (Ip et al., 2021), this is, the greater the dominance of one eye over the other, the higher levels of GABA are detected during monocular stimulation of the dominant eye compared to monocular stimulation of the non-dominant eye (41). While anodal tDCS reduces GABA levels and is therapeutic when applied to both visual cortices (anode located at the occipital midline), cathodal tDCS seems to be effective only when delivered to a single cortex. Thus, bilateral stimulation of V1, which is the anode in one hemisphere and the cathode in the other could result in further decreased GABA levels in the cortex with the anode, as it combines the direct effect of anode decreasing GABA on its target and the indirect effect of cathodal tDCS decreasing interhemispheric inhibitory activity by suppressing the activity in the contralateral V1.

We hypothesized that the bilateral application of tDCS in V1 would improve visual acuity in amblyopic patients. To conduct the first assessment of the effect of bilateral tDCS on visual acuity, in the current study, we investigated the clinical and neurophysiological effects on visual parameters of amblyopic volunteers treated with bilateral tDCS combined with ocular occlusion compared to the effects on a control group of amblyopic volunteers treated with visual occlusion and sham tDCS.

## 2. Materials and methods

### 2.1. Participants and intervention

The study was carried out in accordance with the local regulations and following the ethical standards of the Declaration of Helsinki. All procedures were evaluated and approved by the Scientific or Research Ethics Committee of the Clinical Hospital of the University of Chile under the protocol CECI-HCUCH No. 44-2019. Written informed consent was obtained from all participants.

Twelve adult patients (23–53 years old, 37.6 ± 11.37) with previously diagnosed unilateral amblyopia were recruited in this study from December 2019 to January 2020. Inclusion criteria were amblyopia diagnosis and age between 18 and 65 years old. Exclusion criteria were ophthalmologic disease other than amblyopia, chronic pharmacological therapy, implanted medical device, neurologic disease or surgery history, adverse reaction to tDCS history, pregnancy, inability to give informed consent.

Participants were divided into two groups by simple randomization with an allocation ratio of 1:1 through Optimal Design Software (Michigan, USA). Both participants and research staff were blinded to the group assignment. After eye examination by a strabismus-specialized ophthalmologist, it was found that two participants did not fulfill the criteria for amblyopia diagnosis and were excluded from the study, resulting in *n* = 5 for the bilateral stimulation group (stim group) and *n* = 5 in the sham group. Amblyopia was clinically defined as a difference of BCVA between eyes of at least 0.2 LogMAR, and classified as strabismic, anisometric, or mixed amblyopia.

None of the patients reported previous tDCS treatment, so the blinding process was not affected for the group subjected to sham. For all participants, the refractive correction was always used if needed, including during clinical measurements, EEG recordings, and tDCS sessions. All measurements and data collection were performed in facilities of the Departamento Maria de los Angeles Juricic Tecnología Médica, Universidad de Chile and Servicio de Oftalmología, Hospital Clínico de la Universidad de Chile, Independencia, Chile.

After the eye examination and the ophthalmologic anamnesis were performed, the study was conducted in three stages: baseline measurement of clinical variables (t0), tDCS intervention, and 30 min post-intervention measurement of clinical variables (t1). Patient follow-up was not considered in the design of the current study. The tDCS intervention consisted of the following steps: while sitting in a comfortable chair, participants installed the stimulation sponges and the fellow eye occluded with a patch. The participant was asked to start reading, and the active or sham stimulation protocol was delivered (described below) for 20 min. The participant and the researcher who manipulated the stimulator were blinded to the stimulation protocol applied in each session. After 20 min, the stimulation protocol ended, and the patch and sponges were immediately removed.

### 2.2. Clinical measurements

Specific ophthalmologic examination data were collected before and after tDCS/Sham, including participants’ visual acuity (VA), stereopsis, and contrast sensitivity (CS). Visual acuity was measured with the LogMAR chart at six meters distance and in dim light, using the Nidek CP-770 chart projector (Nidek, Gamagori, Japan) (Bailey and Lovie-Kitchin, 2013). The stereoacuity under natural light was evaluated using the Titmus Stereopsis Test (Stereo Optical Co., Chicago, IL, USA) (Ancona et al., 2014). This test consists of a large-disparity housefly, three series of animals, and nine sets of circles. Contrast sensitivity was measured with the Pelli-Robson contrast sensitivity chart, using OpenTestChart Software\4.1 (OpenOptometry, Thornaby, United Kingdom) (Mäntyjärvi and Laitinen, 2001; Kaur and Gurnani, 2022).

### 2.3. Visual evoked potentials

Visual evoked potentials (VEP) consist of the average EEG signal centered around a stimulus repeated many times. EEG recordings to obtain VEPs were conducted using the Starstim 8 tDCS/EEG device (Neuroelectrics, Barcelona, Spain), using its proprietary software (NIC). EEG signal was acquired and digitized at 500 Hz sample rate and stored. During the recording, a 144 Hz 1ms response display (LG Electronics, Seoul, South Korea) was used for checkerboard pattern stimuli presentation. Synchronization with the NIC recording software was achieved by a script with LabStremingLayer in PsychoPy3 (Peirce et al., 2019). The stimulus for the VEPs was a checkerboard pattern reversing every 500 ms, and 100 reversals were averaged. A visual occlusion patch was used for isolating amblyopic and fellow eye visual stimulation for Visual evoked Potentials. The order of measurement consisted of the fellow eye first, then the amblyopic eye both in pre and post-stimulation. VEPs were constructed offline using the signal from electrode Oz, and Fz as a reference, in concordance with the International Society for Clinical Electrophysiology of Vision (ISCEV) guidelines for visual evoked potentials (Odom et al., 2016). The referenced Oz signal was passband filtered (2–40 Hz) with a second-order default Windowed Sync filter (EEGLab, ERPLab extension, SCCN, San Diego, CA, USA) (Delorme and Makeig, 2004). The resulting signal was divided in 100 periods (–100 to 500 ms around the stimulus) which were averaged to obtain the visual evoked potential—VEP. No artifact rejection was performed as this is optional, according to ISCEV. VEP peak amplitude was used as the main outcome and defined as the difference between the negative peak (N1) between 60 and 110 ms after pattern reversal and the positive peak (P1) between 90 and 160 ms after pattern reversal.

### 2.4. Transcranial direct current stimulation

**FIGURE 1:**
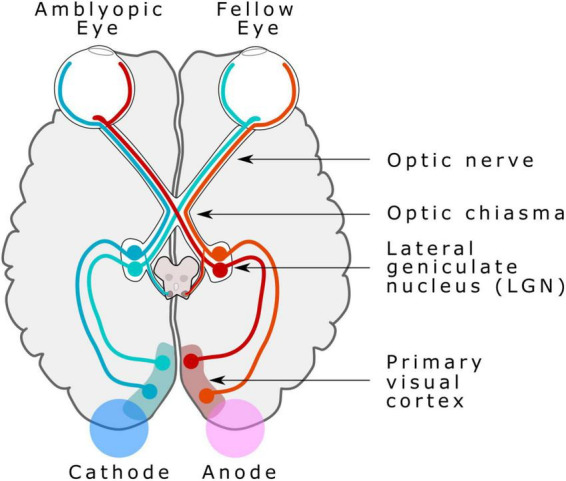
Bilateral transcranial direct current stimulation (tDCS) electrode placement. Anodal tDCS electrode was placed in the contralateral V1 in relation to the amblyopic eye, and a cathodal tDCS electrode was placed in the ipsilateral visual cortex in relation to the amblyopic eye. Image modified from the figure. A simplified schema of the human visual pathway, by Miquel Perello Nieto, licensed under CC BY-SA 4.0.

Two sponges (25 cm^2^ circular area) soaked in saline solution and placed in O1 and O2 (10-20 EEG coordinates system) were used to deliver bilateral tDCS stimulation (2 mA for 20 min, 5 s ramp up and down), driven by a Starstim 8^®^ tDCS device (Neuroelectrics, Barcelona, Spain). The tDCS anode was placed in the contralateral and the cathode in the ipsilateral V1 in relation to the amblyopic eye (Figure 1). Sham stimulation consisted of the same placement and configuration of electrodes as the bilateral active stimulation, but just 30 s of stimulation at 2 mA were delivered at the onset and the offset of the 20 min stimulation period, including the same ramps.

### 2.5. Statistical analysis

GraphPad Prism 6.0 was used for statistical analysis. The changes before and after the intervention (Pre-Post intervention) were subjected to a Mann-Whitney U-test, comparing the bilateral-tDCS Group and the Sham Group. Cohen’s d allowed the quantification of the effect size for significant differences. The critical *p*-value was <0.05. Results are expressed by the mean ± s.e.m.

## 3. Results

The results from a total of 10 participants were included in this study. Recruitment was halted due to the early achievement of the expected outcomes coincident with the mobility restrictions resulting from the COVID-19 pandemic in the country. The mean age among participants was 37.6 (35.2 ± 5.8 stim group and 39.6 ± 4.2 sham group, mean ± s.e.m. in years). History of strabismic amblyopia was found in two participants, anisometric amblyopia in four participants, and mixed amblyopia in four participants. VEP recordings from two participants (one from each group) were lost from a device malfunction. Participants’ characteristics can be examined in Table 1. Secondary or harmful effects due to tDCS were not experienced by participants.

**TABLE 1:** Characteristics of the participants.

tDCS protocol	Age	Gender	Type of amblyopia	Amblyopic eye	Amblyopic eye visual acuity	Fellow eye visual acuity
Bilateral stimulation	26	Male	Mixed	Left	+0.7	–0.1
Bilateral stimulation	30	Female	Strabismic	Right	+0.6	0
Bilateral stimulation	53	Female	Anisometric	Right	+0.5	0
Bilateral stimulation	23	Female	Anisometric	Left	+0.5	0
Bilateral stimulation	45	Female	Mixed	Left	+0.7	0
Sham stimulation	51	Male	Mixed	Right	+0.9	–0.1
Sham stimulation	50	Female	Mixed	Left	+0.3	+0.1
Sham stimulation	23	Male	Anisometric	Left	+1	+0.1
Sham stimulation	34	Female	Strabismic	Right	+1	–0.1
Sham stimulation	38	Male	Anisometric	Right	+0.7	0

Age (years), gender, type of amblyopia, amblyopic eye, amblyopic eye visual acuity and fellow eye visual acuity (LogMAR).

### 3.1. Effect of tDCS on clinical measurements of visual function

**FIGURE 2:**
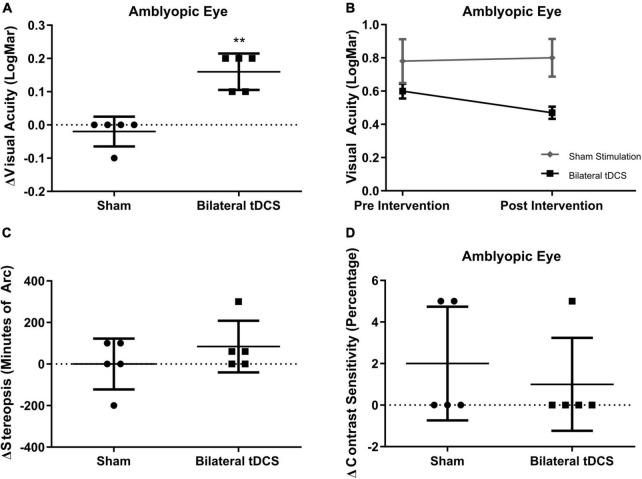
Effects of bilateral-transcranial direct current stimulation (tDCS) on visual acuity, contrast sensitivity and stereopsis. **(A)** Changes in visual acuity of amblyopic eye between groups. **(B)** Pre-Post comparison of visual acuity of amblyopic eye between groups. **(C)** Changes in stereopsis between groups. **(D)** Changes in contrast sensitivity between groups. Mean and standard deviation are shown in **(A,C,D)**. Mean and standard error of mean are shown in **(B)**. For statistical significance analysis Mann-Whithney U-Test was used, **p = 0.0079, Effect Size 2.928 (Cohen’s d). Sham stimulation *n* = 5; Bilateral tDCS *n* = 5.

To determine if bilateral tDCS stimulation improved clinical parameters of visual function, we measured visual acuity, stereopsis, and contrast sensitivity in the participants from the sham and the bilateral stimulation groups. A significant increase in the amblyopic eye’s visual acuity was observed in the bilateral stimulation group, compared to the sham group (0.16 ± 0.025 stim group vs –0.02 ± 0.02 sham group pre-post difference in LogMAR, *p* = 0.0079, d = 2.928) (Figures 2A, B and Supplementary Table 1). No differences were observed in the fellow eye’s visual acuity between groups. Regarding changes in stereopsis (84.00 ± 55.64 stim group vs. 0.0 ± 54.77 sham group, in minutes of arc, *p* = 0.6190, Figure 2C and Supplementary Table 2) and percentage changes in contrast sensitivity (1.250 ± 1.250 stim group vs. 2.000 ± 1.225 sham group, *p* > 0.9999, Figure 2D and Supplementary Table 3), no significant differences were observed between bilateral stimulation and sham group.

### 3.2. Effects of bilateral tDCS on visual evoked potentials

**FIGURE 3:**
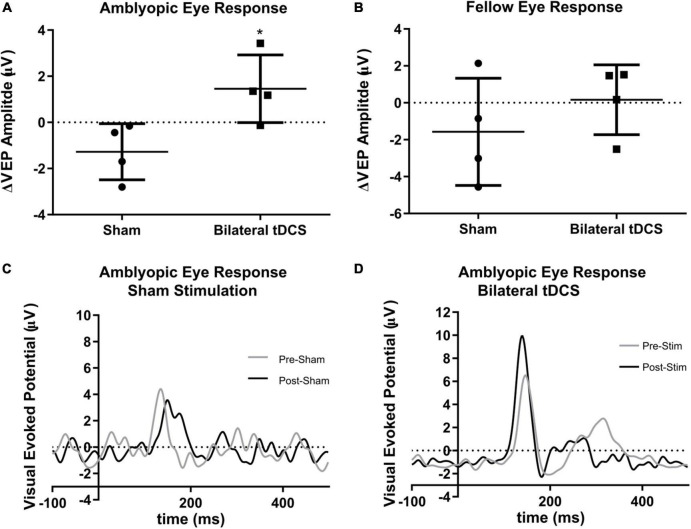
Effect of transcranial direct current stimulation (tDCS) in the visual evoked potential (VEP) amplitude of the fellow and amblyopic eye response. Bilateral tDCS does not change the VEP amplitude of fellow eye response **(A)** but increases the amplitude in the amblyopic eye response **(B)** [Mann-Whithney U-Test *p = 0.0286, Effect Size 2.828 (Cohen’s d), Sham stimulation *n* = 4; Bilateral tDCS *n* = 4, mean and standard deviation shown]. Examples are shown of the amblyopic eye pre and post VEP response from a subject from the Sham **(C)** and the bilateral tDCS **(D)** groups.

To explore whether visual acuity improvement is due to an effect of tDCS over visual cortex activity, we measured the amplitude of visual evoked potentials (VEPs) before and after tDCS. A significant increase in the amplitude of the VEPs of the amblyopic response was observed in the bilateral stimulation group, compared to the sham group (–1.274 ± 0.6088 sham group vs. 1.455 ± 0.7342 bilateral-tDCS group, mean ± s.e.m. in μV, pre-post difference of amplitude of the p100 complex, Mann-Whitney U-test *p* = 0.0286, Cohen’s d = 2.828, Figure 3A and Supplementary Table 4). No differences were observed in the fellow eye response (–1.573 ± 1.452 sham group vs. 0.1618 ± 0.9448 bilateral-tDCS group, mean ± s.e.m. in μV, pre-post difference of amplitude of p100 complex, Mann-Whitney U-test *p* = 0.4857, Figure 3B and Supplementary Table 4). No differences were observed in the latency of the p100 complex. Example VEPs of amblyopic eye response of one subject (pre- and post-stimulation), of both sham and bilateral-tDCS groups, are presented in Figure 3C, D.

## 4. Discussion

To our best knowledge, this is the first study to explore the therapeutic effect of bilateral tDCS in amblyopic patients. Our results show that the combination of anodal tDCS (a-tDCS) in V1 from the hemisphere contralateral to the amblyopic eye and cathodal tDCS (c-tDCS) to V1 of the other hemisphere applied during a single session can improve visual acuity. Our results add to the growing evidence describing tDCS as a potential treatment in adult patients with amblyopia. Previous studies showed similar improvements in visual acuity after applying anodal (Spiegel et al., 2013a) and cathodal unilateral stimulation (Bocci et al., 2018). These studies showed that anodal stimulation to both hemispheres (delivered at the midline) effectively improves amblyopic symptoms, while cathodal applied in the same way is not. Cathodal tDCS is only effective when applied to the left or right of the midline, suggesting that asymmetrical stimulation directed to one hemisphere could be also effective. The present results suggest that enhancing this asymmetrical current stimulation by placing the anode on the contralateral hemisphere might be at least as effective as the other modalities.

Improvement of visual function after tDCS is correlated with an increase of facilitation of the visual evoked potential (VEP) obtained of the amblyopic eye, while no changes or decreased amplitude of the VEP is associated with no improvements in visual function (REFS). Aligned with the previous evidence, we found that VEP of the amblyopic eye is increased after tDCS. In turn, the amplitude of the VEPs in the visual cortex is negatively correlated with the local concentration of GABA (Ding et al., 2016; Bocci et al., 2018). This neurotransmitter seems to be a key element in the mechanisms underlying amblyopic deficits. First, it has been proposed that the maturation of intracortical inhibitory gabaergic neurons determines the closure of the critical period of neuroplasticity that establishes ocular dominance in the visual cortex (Harauzov et al., 2010). Second, pharmacological inhibition of GABA synthesis or its receptors can reactivate neuroplasticity in the visual cortex of adult rats (Sale et al., 2010). Beyond its effect on plasticity, GABA has been implied directly in the intracortical (Liu et al., 2004; Ip et al., 2021) and interhemispheric (Palmer et al., 2012; Restani and Caleo, 2016; Bocci et al., 2018) suppression exerted by the dominant eye over the amblyopic. As the potential for increased plasticity in the human visual system is preserved in adult life (Lunghi et al., 2011; Lo Verde et al., 2017), the decrease in GABA levels by tDCS in the visual cortex where anodal stimulation was performed could be impacting two different aspects: increasing plasticity and preventing or alleviating the suppression exerted by the areas under the control of the dominant eye over the areas or circuits destined to be controlled by the amblyopic eye. Cathodal tDCS on the other hemisphere would suppress the activity of the neurons responsible for transcallosal inhibition, thus relieving interhemispheric suppression.

However, why would the anodal activating effects of tDCS somehow bypass the portions of the cortex under the control of the dominant eye and affect only those areas in which the amblyopic eye is represented? First, healthy cortical elements do not show consistent changes in excitability upon anodal tDCS as observed in the healthy motor cortices (Jonker et al., 2021), second, anodal tDCS applied to healthy cortices or subjects does not cause noticeable improvements (Ding et al., 2016). Taking these two notions, it could be speculated that the therapeutic effects of anodal tDCS are mediated not by increased excitability of the amblyopic visual cortices but by a relief of the suppression normally imposed to them. On the other hand, the selectivity of the cathodal tDCS suppressing effect, which produces a decrease in both GABA and glutamate (Stagg et al., 2009), is only noticeable because it will affect only the active (dominant) cortex and have no effect over the cortex that is already suppressed. Finally, a critical element to consider for the selectivity of both anodal and cathodal effects is the tDCS was performed exclusively during the occlusion of the fellow eye, which means the circuits and cortical areas representing the fellow eye were deprived of natural stimulation, while the areas from the amblyopic eye were stimulated.

Besides the modulation of GABA and glutamate, other mechanisms could contribute to the effect of tDCS. One previous study has shown tDCS to increase the expression of brain-derived neurotrophic factor (BDNF) (Fritsch et al., 2010), which is a remarkably robust, positive modulator of theta-burst induced long-term potentiation (LTP) (Rex et al., 2007). The increased expression of BDNF correlates with improved visual acuity in animal models of amblyopia (Ruiz-Perera et al., 2015). Thus, positive modulation of LTP through increased BDNF is a molecular mechanism other than the GABA/glutamate ratio that could explain the visual acuity improvement obtained in our patients. Likewise, LTP mediated by N-methyl-D-aspartate (NMDA) receptors has been thought to underlie the long-term effects, 48 h post-stimulation shown by Ding et al. (2016). The LTP-like effect of a-tDCS on the motor cortex seems to be favored by the repetition of the stimulation within a time interval of 30 min (Agboada et al., 2020), so a next step in the area of amblyopic visual cortex stimulation would be to develop protocols with multiple sessions, seeking the optimal frequency and number of sessions.

In the present study, contrast sensitivity did not show significant improvement after bilateral tDCS. Previous studies have shown inconsistent changes in this variable (Antal et al., 2001; Kraft et al., 2010; Spiegel et al., 2012; Peters et al., 2013; Costa et al., 2015), probably due to the different techniques used to measure it. Ding et al. (2016) showed a contrast sensitivity improvement but using a Gabor patch test, a different and more sensitive contrast vision assessment than the one used in our study (McAnany and Alexander, 2006; Ding et al., 2016). Despite showing an upward trend, stereopsis was not significantly better in those stimulated with bilateral tDCS (b-tDCS) when compared to controls. However, Titmus test has been reported to be less sensitive than other tests available to quantify stereopsis (Ancona et al., 2014); thus, using more sensitive tests in the future could reveal a significant improvement in this variable, as seen in another study that applied tDCS in patients with amblyopia (Spiegel et al., 2013b). Furthermore, other studies studying treatments for amblyopia have also failed to show significant changes in stereopsis (Pediatric Eye Disease Investigator Group, Holmes et al., 2019). The upward trend observed in the stereopsis of subjects undergoing bilateral tDCS suggests that a higher number of patients recruited in a future study could reveal a significant difference in this variable. Thus, the absence of improvement in stereopsis and contrast vision could be due to the low number of patients and to the low sensitivity of the tests (Titmus test and Pelli-Robson Chart) used to measure these variables.

Although the group with bilateral tDCS showed improved visual acuity after stimulation while the sham group didn’t, the two groups showed baseline differences. The sham group had a higher LogMAR average and a wider range than the stimulation group (see Supplementary Table 2), which might suggest that potential changes due to the intervention in the sham group are obscured because of the higher dispersion of the baseline values in this group. Yet, three participants had zero pre-post LogMAR difference and two participants 0.1, which indicates that although the sham and stimulation group have dissimilar baselines, no changes occurred due to the sham intervention. A relevant limitation of our study is that we didn’t explore glutamate and GABA levels in the visual cortex, which would have been extremely useful considering that the changes of these neurotransmitters are suggested as mechanisms for the modulation of neural activity by tDCS. A tool that could allow us to approach the molecular mechanism by which tDCS modulates visual acuity would be magnetic resonance spectroscopy, which has previously been used to describe changes in neurotransmitter levels after focal application of tDCS in the cerebral cortex (Clark et al., 2011; Kim et al., 2014).

Given the small sample of our study and the fact that the patients were not followed up for longer periods, this is an exploratory study that provides preliminary evidence that bilateral cathodal and anodal tDCS stimulation is a viable option to improve clinical variables of amblyopia in adults. Future studies with a higher number of participants could confirm our overall results by comparing the effectiveness of unilateral anodal, cathodal, and bilateral stimulation in amblyopia and exploring the long-term results of these stimulation protocols.

## Data availability statement

The original contributions presented in this study are included in this article/Supplementary material, further inquiries can be directed to the corresponding author.

## Ethics statement

The studies involving human participants were reviewed and approved by Scientific or Research Ethics Committee of the Clinical Hospital of University of Chile. The patients/participants provided their written informed consent to participate in this study.

## Author contributions

RC-A and LD: original idea and conceptualization. RC-A, JM, LD, and RF-F: study design and stimulation protocol. RF-F, MA, PM, and MV: formal analysis and concept proofing. MV: patient recruitment and diagnosis confirmation. LD and IP-R: measurements. RC-A: stimulation execution. RC-A, IP-R, and JM: statistical and data analysis. LD and RC-A: writing—original draft preparation. MA, RF-F, IP-R, and JM: writing—review and editing. RF-F, PM, and MV: supervision. All authors contributed to the article and approved the submitted version.
