# Supplementary material for: Combined therapy of bilateral transcranial direct current stimulation and ocular occlusion improves visual function in adults with amblyopia, a randomized pilot study
Source: Front Hum Neurosci. 2023 Feb 3;17:1056432. doi: 10.3389/fnhum.2023.1056432 (PMC9936073; doi:10.3389/fnhum.2023.1056432)
Supplement: Supplementary file 1 [file Table_1.DOCX]

Supplementary Material for “Combined therapy of bilateral transcranial direct current stimulation and ocular occlusion improves visual function in adults with amblyopia, a randomized pilot study.”

# Supplementary Table 1.

Visual Acuity, and differences pre-post stimulation. All values are in LogMar. The order is the same as in Table 1 from main text.

| Group | Visual Acuity Amblyopic Eye, Pre-Stimulation | Visual Acuity Amblyopic Eye, Post-Stimulation | Change in Visual Acuity |
| --- | --- | --- | --- |
| Stim | 0.7 | 0.6 | 0.1 |
| Stim | 0.6 | 0.4 | 0.2 |
| Stim | 0.5 | 0.4 | 0.1 |
| Stim | 0.5 | 0.45 | 0.05 |
| Stim | 0.7 | 0.5 | 0.2 |
| Sham | 0.9 | 0.9 | 0 |
| Sham | 0.3 | 0.4 | -0.1 |
| Sham | 1 | 1 | 0 |
| Sham | 1 | 1 | 0 |
| Sham | 0.7 | 0.7 | 0 |

Stim: 0.16 ± 0.02 (mean ± standard error of the mean)

Sham: -0.02 ± 0.02 (mean ± standard error of the mean)

# Supplementary Table 2

Stereopsis, and differences pre-post stimulation. All values in minutes of arc. The order is the same as in Table 1 from main text.

| Group | Stereopsis, Pre-Stimulation | Stereopsis, Post-Stimulation | Change in Stereopsis |
| --- | --- | --- | --- |
| Stim | 400 | 100 | 300 |
| Stim | 400 | 400 | 0 |
| Stim | 100 | 100 | 0 |
| Stim | 200 | 140 | 60 |
| Stim | 200 | 140 | 60 |
| Sham | 400 | 400 | 0 |
| Sham | 400 | 300 | 100 |
| Sham | 400 | 400 | 0 |
| Sham | 400 | 300 | 100 |
| Sham | 200 | 400 | -200 |

Stim: 84.0 ± 55.6 (mean ± standard error of the mean)

Sham: 0.0 ± 54.8 (mean ± standard error of the mean)

# Supplementary Table 3.

Visual Sensitivity, and differences pre-post stimulation. All values in percentage of visual sensitivity. The order is the same as in Table 1 from main text.

| Group | Visual Sensitivity Amblyopic Eye, Pre-Stimulation | Visual Sensitivity Amblyopic Eye, Post-Stimulation | Change in Visual Sensitivity |
| --- | --- | --- | --- |
| Stim | 5% | 5% | 0 |
| Stim | 5% | 5% | 0 |
| Stim | 10% | 10% | 0 |
| Stim | 5% | 5% | 0 |
| Stim | 10% | 5% | 5 |
| Sham | 10% | 5% | 5% |
| Sham | 10% | 10% | 0 |
| Sham | 10% | 5% | 5 |
| Sham | 5% | 5% | 0 |
| Sham | 15% | 15% | 0 |

Stim: 2 ± 55.6 (mean ± standard error of the mean)

Sham: 2 ± 54.8 (mean ± standard error of the mean)

# Supplementary Table 4.

Visual Evoked Potentials, and differences pre-post stimulation. All values are in mV.

| VEP Amplitude Pre-Stimulation | VEP Amplitude Post-Stimulation | Difference in VEP Amplitude |
| --- | --- | --- |
| Amblyopic Eye Response, Stimulated Group | | |
| 7.003651629 | 8.180722323 | 1.177070695 |
| 7.119827271 | 10.54220731 | 3.422380043 |
| 12.16318766 | 13.51359758 | 1.350409922 |
| 4.830462831 | 4.701210561 | -0.12925227 |
| Amblyopic Eye Response, Sham Group | | |
| 5.784127515 | 4.090352415 | -1.6937751 |
| 6.372510505 | 3.571254692 | -2.801255814 |
| 4.909533424 | 4.468620377 | -0.440913046 |
| 4.154492706 | 3.996078645 | -0.15841406 |
| Fellow Eye Response, Stimulated Group | | |
| 6.57689004 | 8.093624847 | 1.516734807 |
| 10.63459223 | 12.10565494 | 1.471062708 |
| 18.12940409 | 18.30317241 | 0.173768323 |
| 8.210430155 | 5.696253459 | -2.514176696 |
| Fellow Eye Response, Sham Group | | |
| 11.22980314 | 6.666084174 | -4.563718969 |
| 13.00029593 | 12.14909449 | -0.851201433 |
| 14.50309476 | 16.64070214 | 2.137607382 |
| 8.501033744 | 5.486200121 | -3.014833624 |

Amblyopic Eye Response

Sham group: -1.274 ± 0.6088 µV (mean ± s.e.m.)

Bilateral-tDCS group: 1.455 ± 0.7342 µV (mean ± s.e.m.)

Fellow Eye Response

Sham group: -1.573 ± 1.452 µV, mean ± s.e.m.

bilateral-tDCS group : 0.1618 ± 0.9448 µV, mean ± s.e.m.
